# Identification of the Genetic Characteristics of Copy Number Variation Regions in Diverse Goat Populations

**DOI:** 10.3390/genes17060627

**Published:** 2026-05-30

**Authors:** Wenze Li, Yixin Su, Can Liu, Xiaochun Yan, Qi Lv, Rui Su

**Affiliations:** 1 College of Animal Science, Inner Mongolia Agricultural University, Hohhot 010018, Chinayixinwink@126.com (Y.S.); 15847756571@163.com (C.L.); yanxiaochunan@163.com (X.Y.); lvqi1202@imau.edu.cn (Q.L.); 2 Sino-Arabian Joint Laboratory of Sheep and Goat Germplasm Innovation, Hohhot 010018, China; 3 Inner Mongolia Key Laboratory of Sheep & Goat Genetics Breeding and Reproduction, Hohhot 010018, China

**Keywords:** goats, whole genome resequencing, copy number variation region, selection signature

## Abstract

Background: Copy number variation (CNV) is an important class of structural variations (SVs) that contribute to phenotypic diversity and environmental adaptation in animals. However, large-scale population-level analyses of CNVs in goats remain limited. This study aimed to comprehensively characterize CNVs and explore their potential roles in economically important traits in Chinese goat populations. Methods: Whole-genome resequencing data from 151 individuals representing 17 Chinese goat breeds were analyzed. CNV regions (CNVRs) were identified across the genome, followed by gene annotation, functional enrichment analysis, and population differentiation analysis based on V_ST_. Results: A total of 5636 CNVRs were identified from 151 individuals of 17 goat breeds, including 1365 duplication CNVRs, 4241 deletion CNVRs, and 30 both CNVRs. These CNVRs collectively spanned 2.38% of the goat genome. A total of 912 protein-coding genes overlapped with these CNVRs. After Bonferroni correction, GO enrichment analysis showed that these genes were significantly enriched in terms related to transmembrane transport, cell projection, ion binding, and ATP binding. Population differentiation analysis identified several CNVR-associated candidate genes with potential relevance to production or adaptive traits, including *ABCC4*, *APOL3*, *EXOC3L4*, *ERG*, *B4GALT1*, and *FTH*. Conclusions: This study provides a comprehensive CNVR map of Chinese goat populations and offers insights into the genetic basis of economically important traits, contributing to future genetic improvement and breeding strategies in goats.

## 1. Introduction

The goat (*Capra hircus*) is one of the earliest domesticated livestock species, with domestication dating back to the early Neolithic era (approximately 11,000 years before present, YBP) in the Fertile Crescent [1]. Driven by human demand for specific economic traits, goats have been subjected to long-term artificial selection, resulting in breeds specialized for meat, dairy, wool, cashmere, and leather production [2]. In addition, environmental variation, geographical isolation, and human migration have all contributed to goat selection and population differentiation. After long-term domestication and selection, genomic diversity among goat breeds has increased, leading to pronounced phenotypic variation. The major genetic sources of trait variation include single nucleotide polymorphisms (SNPs), insertions and deletions (InDels), structural variations (SVs), and copy number variations (CNVs) [3]. However, studies investigating the molecular mechanisms underlying economically important traits in goats have largely focused on SNPs, while CNV-based analyses remain relatively limited.

CNV is a widespread form of SV in animal genomes, typically ranging from ~50 bp to several megabases, and arising mainly from deletions and duplications of DNA segments [3]. CNVs can substantially contribute to phenotypic variation in plants and animals [4]. Compared with SNPs and indels, CNVs often span larger genomic regions, exhibit higher per-site mutation rates, and encompass more complex variants, making them valuable for studies of animal genetics and breeding. With advances in omics and high-throughput sequencing, genome-wide CNV detection has become an important strategy for dissecting the genetic basis of animal phenotypes and population variation. The CNVs detected between different individuals have some overlapping regions. The overlapping CNVs are integrated into a CNV region (CNVR). The study of animal diversity mainly focuses on the CNV regions (CNVRs), which make a great contribution to the understanding of animal evolution, adaptation and trait formation mechanisms [5].

There are roughly two methods commonly used in animals and plants to detect CNVs. One approach is to detect unknown CNVs on a genome-wide scale, with the main methods being chip sequencing and DNA sequencing [6]. DNA sequencing mainly includes Whole Genome Sequencing (WGS) and Long Read Sequencing (Third-Generation Sequencing, TGS). Another method is to detect the identified target CNVs throughout the entire genome using hybridization and PCR techniques. The methods based on hybridization technology include DNA Fluorescence in Situ Hybridization (FISH) and Multiplex Amplifiable Probe Hybridization (MAPH) [7], etc., while the methods based on PCR technology include quantitative PCR (qPCR)and digital PCR (dPCR) [8]. Recently, next-generation genome sequencing technologies have been continuously used to detect the genome-wide CNVs of livestock [9]. However, there are currently relatively few studies on CNVs in various goat breeds of China and even fewer studies on CNVs in cashmere goats.

In the present study, we performed a genome-wide CNV analysis using genomic resequencing data in 17 Chinese goat breeds. The purpose of this study was to generate a comprehensive CNVR landscape in Chinese goats, compare CNVR diversity among cashmere, meat, and dairy goats, and explore candidate CNVRs potentially involved in the differentiation of cashmere goats. The results of this study provide data support for analyzing the trait differentiation of cashmere goats, meat goats and dairy goats.

## 2. Materials and Methods

### 2.1. Sample Collection

Tissue samples were collected from 151 goats representing 17 Chinese goat breeds from sixteen regions in eleven provinces of China (Table S1). According to their primary production purposes, these breeds were classified into three production categories: cashmere goats, meat goats, and dairy goats. The cashmere-goat group included 124 individuals from 14 breeds, including Arbas cashmere goats (AEBS, *n* = 9), Alashan cashmere goats (ALS, *n* = 7), Erlangshan cashmere goats (ELS, *n* = 9), Ujimqin white goats (WZMQ, *n* = 9), Chaidamu goats (CDM, *n* = 9), Cuoqin purple cashmere goats (CQ, *n* = 9), Hanshan cashmere goats (HS, *n* = 9), Hexi cashmere goats (HX, *n* = 9), Jining grey goats (JN, *n* = 9), Jinlan cashmere goats (JL, *n* = 9), Liaoning cashmere goats (LN, *n* = 9), Lazi grey cashmere goats (LZ, *n* = 9), Shaanbei white cashmere goats (SBW, *n* = 9), and Xizang Northwest white cashmere goats (ZXB, *n* = 9). The meat-goat group included 18 individuals from two breeds, Hainan black goats (HN, *n* = 9) and Yunshang goats (YS, *n* = 9), whereas the dairy-goat group included 9 Laoshan dairy goats (LS, *n* = 9). The sampled animals included 69 males and 82 females. Samples were collected from original breeding farms or representative conservation populations. Genomic DNA was extracted from tissue samples using the standard phenol–chloroform method. DNA integrity was assessed by agarose gel electrophoresis, and DNA concentration and purity were evaluated using a NanoDrop spectrophotometer (Thermo Fisher Scientific, Waltham, MA, USA) before library construction. Paired-end sequencing (150 bp) was performed using the MGISEQ-2000 platform (MGI Tech Co., Ltd., Shenzhen, China).

### 2.2. Sequence Alignment

After removing low-quality reads (including reads with more than 10% unidentified nucleotides (N), adaptor contamination, more than 50% of bases with Phred quality < 5, and putative PCR duplicates), a total of 12.85 Tb of clean data were obtained (Table S1). And the average sequencing depth was approximately 30.40×.

The remaining high-quality paired-end reads were aligned to the cashmere goat reference genome (GCA_040822015.1) [10] using BWA v0.7.8 with the parameters “mem -t 4 -k 32 -M”, where “-k” specifies the minimum seed length and “-M” marks shorter split alignments as secondary alignments. To reduce bias introduced by PCR amplification, duplicated reads were marked and removed using Picard v3.20. Because read-depth-based CNV detection is sensitive to sequencing-depth fluctuation and local coverage bias, the coefficient of variation (CV) of normalized read depth across genomic windows was evaluated for each individual. All samples showed acceptable CV values (<0.5) and were retained for downstream CNVR detection. In addition, GC-bias correction, correction for highly similar genomic regions, correlation-based merging of adjacent windows, silhouette score filtering, and length filtering were applied to reduce the potential influence of depth variation on CNVR calling.

### 2.3. Determination of CNVRs

CNVcaller v0.0.2024.01.18 [11] was used to identify CNVRs in the 151 goats. This software was selected because it is specifically designed for population-scale CNV detection from whole-genome resequencing data in livestock and other non-model species. Unlike tools that detect CNVs separately in each individual, CNVcaller integrates read-depth information across all individuals to define shared CNVRs and performs correction for GC bias and highly similar genomic regions. Therefore, it is suitable for detecting population-level CNVRs in large-scale resequencing datasets. First, to construct the *C. hircus* reference database, the cashmere goat reference genome was segmented into overlapping windows of 800 bp [11]. Second, the read count in each window was calculated, and reads with high similarity (≥97%) were merged into autosomal segments. Third, the GC bias was used to standardize the copy number in each window, and it was used to classify the different genotypes of each sample. Finally, CNVRs were filtered using the following criteria: -f 0.1, -h 3, and -r 0.2. CNVRs were defined by aggregating adjacent candidate CNV windows across individuals. Regions were retained when more than 10% of individuals exhibited copy number variation (-f 0.1) or at least three individuals showed homozygous variation (-h 3). Adjacent windows were merged into CNVRs when their copy number profiles were significantly correlated (r > 0.2), ensuring biological consistency. To ensure accurate genotyping, CNVRs with low clustering quality were excluded. The silhouette score was used to evaluate the separation of copy number states after genotyping, and only CNVRs with silhouette scores greater than 0.6 were retained, indicating reliable clustering of copy number genotypes. CNVRs were further filtered based on length following previous CNVcaller-based studies. Specifically, CNVRs longer than 50 kb for deletions and both types, and longer than 500 kb for duplications, were excluded [12]. These length filters were applied to reduce potential false-positive signals caused by extremely long read-depth fluctuations, repetitive regions, mapping ambiguity, or assembly-related artifacts.

### 2.4. PCA and Phylogenetic Analysis

Principal component analysis (PCA) was performed to assess population structure and genetic relationships among goat populations. Only high-confidence CNVRs that passed the filtering criteria described above were used for PCA and phylogenetic analysis. VCF files were converted to PLINK format using VCFtools v0.1.17, and PCA was conducted using PLINK v1.90. The results were visualized using the R v4.2.2. A pairwise genetic distance matrix based on CNVR genotypes was calculated using PLINK v1.90, and a neighbor-joining (NJ) tree was constructed using Phylip v.3.696. The distance matrix was converted into Phylip format using a Perl v5.42.0, and the tree was visualized using iTOL (https://itol.embl.de/, accessed on 20 June 2025). The NJ tree was used to visualize CNVR-based population relationships.

### 2.5. CNVRs Annotation and Enrichment Analysis

The identified CNVRs were annotated using ANNOVAR v2020-06-08, and genes overlapping with CNVRs were identified using BEDTools v2.26.0. Functional enrichment analysis of CNVR-overlapping genes was performed using DAVID (https://david.ncifcrf.gov/, accessed on 20 June 2025) [13]. GO terms included biological process, cellular component, and molecular function categories. To account for multiple testing, *p*-values were adjusted using the Benjamini–Hochberg method. Terms with adjusted *p*-value < 0.05 were considered statistically significant.

### 2.6. Comparison of CNVRs Between Different Groups

To investigate CNVR differentiation related to production purpose, we first split the 151 goats into cashmere (124 individuals from 14 breeds) and non-cashmere (27 individuals from three breeds: 18 meat, 9 dairy) groups. The non-cashmere group was used as a reference because both meat and dairy goats lack cashmere production. This comparison was designed to identify CNVRs showing broad differentiation between cashmere-producing and non-cashmere-producing goats. However, given the distinct genetic backgrounds and production traits of meat versus dairy goats, we also performed pairwise V_ST_ analysis across the three production categories (cashmere vs. meat, cashmere vs. dairy, meat vs. dairy). This allowed us to test whether candidate CNVRs from the broad comparison were also supported in more specific contrasts. V_ST_ was calculated between these groups to identify differentiated CNVRs. V_ST_ is an index to measure the differentiation of CNV between different populations by calculating the variance of copy number, similar to the concept of F_ST_, which can be used to identify some highly differentiated regions, and it ranges from 0 (undifferentiated) to 1 (population-specific) [14]. V_ST_ was calculated as V_ST_ = (V_T_ − V_S_)/V_T_, where V_T_ represents the total variance across all individuals and V_S_ is the average within-population variance weighted by population size [12]. Following previous livestock CNVR studies, the top 2% of V_ST_ values were selected as candidate highly differentiated CNVRs [15]. Genes overlapping these CNVRs were subsequently annotated as candidate genes. For candidate genes overlapping highly differentiated CNVRs, copy number differences between cashmere and non-cashmere goats were compared using the Wilcoxon rank-sum test. *p*-value < 0.05 was considered statistically significant.

## 3. Results

### 3.1. Number and Distribution of CNVRs

**Figure 1:**
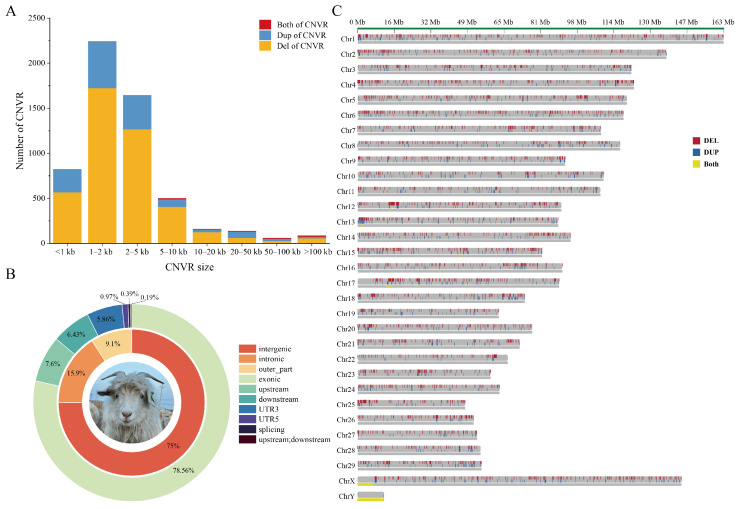
Genomic diversity and distribution of CNVR in 151 goats. (**A**) The length of the detected CNVRs. (**B**) Annotation of CNVRs. The inner circle indicates the intronic region, intergenic region, and the remaining set of functional regions (outer_part). The outer circle includes exonic; upstream; downstream; UTR3; UTR5; splicing; upstream and downstream. (**C**) The chromosome distribution of CNVRs. The locations with different colors represent duplication (blue), deletion (red), and both duplication and deletion (yellow).

CNVcaller was used to identify CNVRs among individuals based on the cashmere goat reference genome (GCA_040822015.1). A total of 5636 CNVRs, including duplications, deletions, and both types, were classified into different length groups (Figure 1A). There were 1365 duplication CNVRs, 4241 deletion CNVRs, and 30 both CNVRs (Table S2). CNVRs of 2–5 kb were the most abundant, accounting for 39.70% of all detected CNVRs. After merging adjacent candidate CNV windows into non-redundant CNVRs, the final CNVR set spanned 65,878,733 bp, covering approximately 2.38% of the goat reference genome (65,878,733/2,762,439,668). Approximately 75% of CNVRs were located in intergenic regions, followed by intronic regions (15.90%). Only 7.15% of CNVRs were located in exonic regions (Figure 1B; Table S3). CNVRs were randomly distributed across chromosomes in terms of both number and length (Figure 1C and Figure S1). The predominance of CNVRs in intergenic regions suggests that most CNVs may have limited direct effects on coding sequences, while those located in exonic regions may have stronger functional consequences.

### 3.2. Population Structure

**Figure 2:**
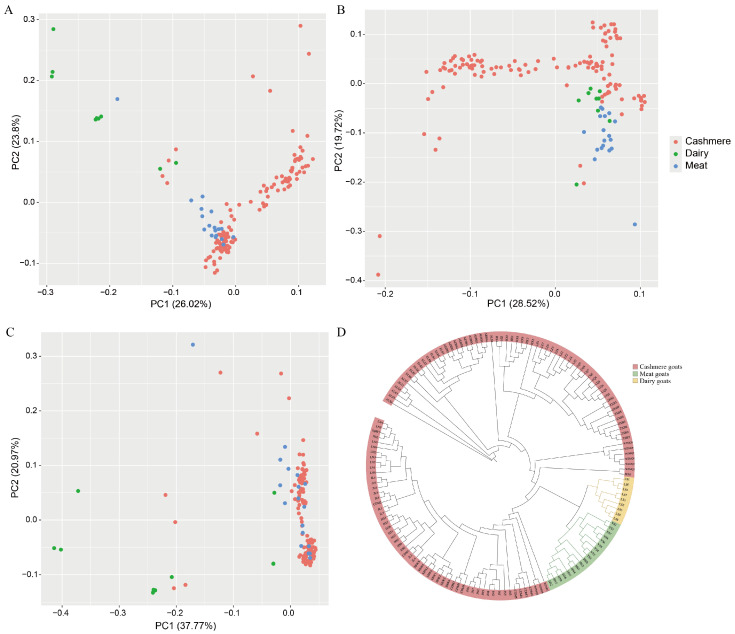
Population structure of 151 Chinese goats based on CNVR genotypes. (**A**) The principal component analysis (PCA) based on all CNVRs. The percentages shown on the PC1 and PC2 axes indicate the proportion of variance explained by each principal component. (**B**) PCA based on deletion CNVRs. (**C**) PCA based on duplication CNVRs. (**D**) Neighbor-joining tree constructed from CNVR-based genetic distance among 17 goat breeds.

With the effect of balancing selection, abundant polymorphisms of the genomic CNVR are found in Chinese goats. Principal component analysis (PCA) was performed to compare cashmere goats and non-cashmere goats based on total CNVRs (Figure 2A), deletions (Figure 2B), and duplications (Figure 2C). For total CNVRs, the first two principal components (PC1 and PC2) explained 26.02% and 23.8% of the total variance. Cashmere goats could be roughly separated from meat and dairy goats, although some individuals overlapped. The PCA results based on deletions were similar to those obtained from the total CNVR dataset. In contrast, duplications were unable to clearly separate cashmere goats from meat goats. Notably, both total CNVRs and deletions could distinguish Lazi gray cashmere goats from other cashmere goat populations, whereas other breeds showed overlapping patterns (Figures S2–S4). This may be related to the specific geographic environment of Lazi gray cashmere goats, which inhabit high-altitude regions (~4000 m) in Shigatse, Xizang Autonomous Region, as well as relatively low selective pressure leading to the accumulation of deletions.

These results suggest that artificial selection has influenced CNVR diversity among goat breeds during domestication. The NJ tree based on CNVR genotypes showed a clustering pattern broadly consistent with the PCA results. Meat and dairy goats tended to cluster closer to each other, whereas cashmere goat populations showed relatively broader differentiation. Some breeds also showed clustering patterns related to their geographic origins or breeding backgrounds.

### 3.3. Annotation of CNVRs

**Figure 3:**
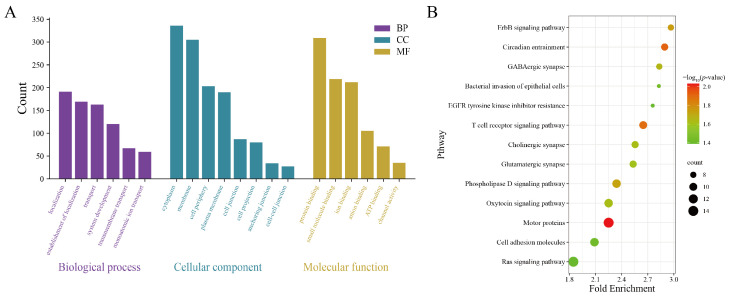
Functional enrichment of CNVR-associated genes. (**A**) GO analysis of CNVR-overlapping genes. (**B**) KEGG analysis of CNVR-overlapping genes.

The 5636 identified CNVRs were annotated using BEDTools v2.26.0. A total of 912 protein-coding genes were annotated. Functional enrichment analysis was performed for these CNVR-overlapping genes. After Bonferroni correction, these genes were significantly enriched in 7 biological process terms, 11 cellular component terms, and 13 molecular function terms (adjusted *p*-value < 0.05; Figure 3). Representative enriched terms included transmembrane transport (GO:0055085, adjusted *p*-value = 0.013), system development (GO:0048731, adjusted *p*-value = 0.048), cell projection (GO:0042995, adjusted *p*-value = 0.0034), cell–cell junction (GO:0005911, adjusted *p*-value = 0.012), ion binding (GO:0043167, adjusted *p*-value = 0.00034), and ATP binding (GO:0005524, adjusted *p*-value = 0.015). KEGG pathway analysis was used to provide a pathway-level functional overview of CNVR-overlapping genes. Several pathways showed nominal enrichment, including Motor proteins, Circadian entrainment, T cell receptor signaling pathway, and Oxytocin signaling pathway.

In addition, QTL data were obtained from the Goat QTL database. Genomic coordinates were converted to the reference genome using CrossMap (v0.6.5), and overlap between QTLs and CNVRs was assessed. Only one QTL (QTL_ID = 285731; chr13: 69,826,471–69,826,475) overlapped with a CNVR associated with chest width (chr13: 69,690,001–69,978,200).

### 3.4. Differentiated CNVRs Between Cashmere Goats and Other Goat Breeds

**Figure 4:**
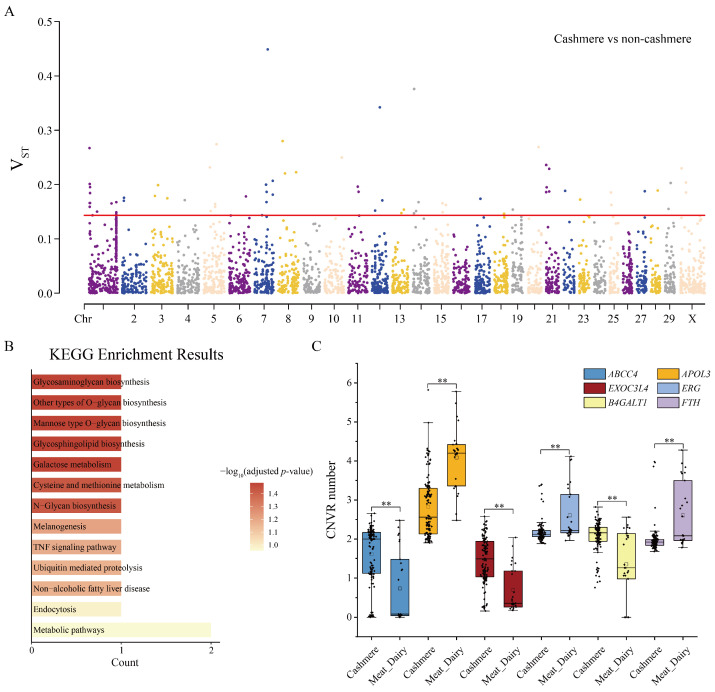
Genome-wide V_ST_ analysis of CNVRs. (**A**) Manhattan plot of V_ST_ value plots between cashmere goats and non-cashmere goats. The red line represents the top 2% of V_ST_ value. (**B**) Enrichment pathways between cashmere goats and non-cashmere goats. (**C**) CNVR comparison of representative candidate genes between cashmere and non-cashmere goats. Different colors represent different candidate genes. Boxes indicate the interquartile range, the horizontal line within each box represents the median, whiskers indicate the data range, and black dots represent individual samples. Statistical significance was evaluated using the Wilcoxon rank-sum test. * *p*-value < 0.05; ** *p*-value < 0.01.

We calculated the V_ST_ between cashmere goats and non-cashmere goats (Table S4). Using the top 2% (98th percentile) of V_ST_ values as the threshold, a total of 15 candidate genes were identified (Figure 4A, Table S5). The distribution of V_ST_ values across the genome revealed a small subset of highly differentiated CNVRs, indicating that most CNVs are shared across populations, while only a few regions are under strong selection. Functional annotation showed that the selection signals between cashmere goats and non-cashmere goats were enriched in galactose metabolism and cysteine and methionine metabolism (Figure 4B; Table S6). By performing a literature search (https://www.ncbi.nlm.nih.gov/, accessed on 10 July 2025) and GeneCards database search (https://www.genecards.org/, accessed on 12 July 2025) on the functions of the genes, we screened hair growth and development (*ABCC4*) [16], growth (*APOL3*) [17], immunity (*EXOC3L4*, *ERG*) [18], lactation (*B4GALT1* [19]), and adaptation (*FTH*) [20]. The copy numbers of these candidate genes were all significantly different in cashmere goats and no- cashmere goats (Figure 4C, Table 1). The identified candidate genes showed clear differences in copy number between groups, suggesting that gene dosage effects may contribute to phenotypic divergence.

Because the non-cashmere group consisted of both meat and dairy goats, we further performed pairwise V_ST_ analyses among the three production categories to evaluate the potential influence of this grouping strategy. Between cashmere goats and meat goats, a total of 14 candidate genes exceeding the threshold were identified (Figure S5, Tables S7 and S8), including *APOL3* and *B4GALT1*. Between cashmere goats and dairy goats, 3 candidate genes exceeding the threshold were identified (Figure S6, Tables S9 and S10), including *ERG*. Between meat goats and dairy goats, 12 candidate genes exceeding the threshold were identified (Figure S7, Tables S11 and S12), including *ERG*.

## 4. Discussion

Goats are among the earliest domesticated livestock species and are characterized by high adaptability and reproductive capacity. Based on their economic uses, they are typically classified into cashmere, meat, and dairy goats. CNVs can alter gene dosage and influence gene expression, thereby contributing to phenotypic variation and economically important traits [21]. In this study, we identified 5636 CNVRs across 17 Chinese goat breeds using whole-genome resequencing data, including 1365 duplication CNVRs, 4241 deletion CNVRs, and 30 both CNVRs. The total length of CNVRs covered 2.38% (65,878,733 bp) of the reference genome in goat, whereas it represented 1.2% in pig (*Sus scrofa*) and 0.3% in sheep (*Ovis aries*) [22,23]. In addition, it was found that about 2.4% of CNVR was present in the African goat [24], which is similar to the results of the present study. Different sample sizes, different detection methods and different versions of the reference genome may result in inconsistencies in the number and length of CNVRs detected. The *C*. *hircus* reference genome used in this study was the male cashmere goat T2T reference genome assembled by the team at an early stage, and the Y chromosome was fully assembled. The samples used in this study included 69 rams and 82 ewes, and the CNVR of these 151 goats was detected. It was found that there were 2 CNVRs on the Y chromosome of 69 rams, and the copy number of CNVRs on each individual was 1. It is speculated that these regions are gender related. When using CNVcaller v0.0.2024.01.18 for CNVR detection, we define a window as a candidate copy number variation window when there is a significant difference (heterozygous deletion or heterozygous duplication) between the absolute copy number of an individual exceeding 0.1 and the normal copy number (“1”) [11]. At the same time, the Y chromosome usually contains a large number of repetitive sequences and palindrome structures, which may cause the Y chromosome to be classified as a variation region during CNVR detection. Therefore, the two CNVRs on the Y chromosome in the CNVR map constructed by this study almost cover the entire chromosome.

We conducted a population genetics study on Chinese goat breeds based on the identified CNVRs. PCA results revealed that cashmere goats could be broadly separated from meat and dairy goats, but some individuals showed overlapping clustering patterns. In contrast, individuals of different breeds for the same purpose showed admixture and were not distinguished by breed (Figures S3–S5), which may be caused by the small differences in PCA results produced by the identified CNVRs among the 17 Chinese goat breeds. It has been shown that CNVRs have less data to genotype compared to snp, making it difficult for them to achieve accurate clustering between breeds and within populations [25].

Changes in the CNVR can result in deletions or duplications of genes, and these changes can alter gene expression [12]. Therefore, annotating genes overlapping CNVRs is important for understanding their potential biological relevance. In this study, GO enrichment analysis showed that CNVR-overlapping genes were significantly enriched in terms related to transmembrane transport, membrane-associated components, cell junctions, ion binding, and ATP binding after Bonferroni correction. These results suggest that CNVRs in Chinese goats may preferentially affect genes involved in membrane structure, molecular binding, and transport-related processes. KEGG pathway analysis provided additional pathway-level information, with several pathways showing nominal enrichment, including Motor proteins, Circadian entrainment, T cell receptor signaling pathway, and Oxytocin signaling pathway. However, these KEGG pathways did not remain significant after multiple-testing correction and should therefore be interpreted only as potential functional clues.

In this study, the top 2% of V_ST_ values were used as an empirical threshold to identify CNVRs with marked differentiation between groups, following previous livestock CNV studies. Because V_ST_ is a descriptive measure of copy number differentiation rather than a formal association test, these CNVRs should be regarded as candidate regions. To minimize potential false-positive interpretation, only high-confidence CNVRs passing strict quality-control filters were included, and the candidate genes were further evaluated in the context of pairwise group comparisons, functional annotation, and previous biological evidence. We compared the differential CNVRs between cashmere goats and non-cashmere goats (meat and dairy goats) using the V_ST_ method, and candidate genes including *ABCC4*, *APOL3*, *EXOC3L4*, *ERG*, *B4GALT1*, and *FTH* were identified from the top 2% regions. The copy number of these gene regions differed significantly between cashmere goats and non-cashmere goats.

ATP-binding cassette sub-family C member 4 (*ABCC4*) belongs to the adenosine triphosphate-binding cassette (ABC) transporter family members. Many members of the ATP family are involved in the regulation of dermal hair follicle stem cells, among which *ABCC4* is widely expressed in hair follicles. It has been shown that the ABC transporter protein family protects epithelial hair follicle stem cells (eHFSCs) epithelial hair follicle stem cells from chemotherapy damage, which means that increasing the expression of certain ABC transporter proteins in eHFSCs may decrease chemotherapy-induced alopecia. In studies related to goats, *ABCC4* has been shown to be associated with the goat immune system and helps goats adapt to their environment [26]. These findings suggest that *ABCC4* may represent a candidate gene for further investigation of hair follicle-related biological processes in cashmere goats.

Cashmere goats, meat goats, and dairy goats differ in body size, production purpose, and management practices. Previous studies have shown that CNV in *APOL3* is significantly associated with growth traits, such as hip height and body length in cattle [17,27]. The CNVR involving *APOL3* may provide a potential clue for production-type differentiation in goats. In addition, due to different living environments, each goat breed may have different disease resistance for better adaptation to the local environment. Previous studies have implicated *EXOC3L4* in immune-related or inflammatory processes in livestock and other mammals [28]. Both CNVR and SNP contained within the *EXOC3L4* gene have been reported to be associated with immune diseases in goats [29]. ETS Transcription Factor ERG (*ERG*) gene encodes a member of the erythroblast transformation-specific (ETS) family of transcription factors. All members of this family are key regulators of embryonic development, cell proliferation, differentiation, angiogenesis, inflammation, and apoptosis. ERG proteins are expressed in endothelial cells and are involved in physiological processes such as angiogenesis and hematopoiesis [30]. They also regulate endothelial homeostasis and inhibit the expression of pro-inflammatory genes [31]. This gene was significantly labeled in V_ST_ analyses in both cashmere and dairy goats.

Dairy production trait is the most important economic trait of dairy goats. Beta-1,4-Galactosyltransferase 1 (*B4GALT1*) encodes a key enzyme involved in lactose synthesis and is considered an important candidate gene for milk production traits [19]. By studying the expression of *B4GALT1* at different lactation periods in dairy goat breeds with different milk yields, it was found that the expression of *B4GALT1* was positively correlated with goat lactation [32]. In addition, goats are mainly found in highland, hilly and mountainous environments, and have the advantages of strong adaptability and good disease resistance compared with other livestock. The ferritin heavy/heart chain (*FTH*) gene is an important factor in the regulation of redox homeostasis, energy consumption and maintenance of adipose tissue homeostasis. It has been reported that deletion of the *FTH* gene not only leads to a decrease in body temperature, but also reduces the area of white adipose tissue and brown adipose tissue, thereby disrupting homeostasis [20]. In addition, *FTH* supports mitochondrial function by regulating cellular iron content [33], thereby responding to the adaptive challenges that the environment imposes on plants and animals. This suggests that CNVRs involving *FTH* may be relevant to environmental adaptation, but their functional effects need to be confirmed in future studies.

We acknowledge that this study has several limitations. First, the CNVRs identified in this study were inferred from read-depth-based whole-genome resequencing data and were not experimentally validated. Although stringent quality-control filters were applied, future validation of representative CNVRs, especially those overlapping *ABCC4*, *APOL3*, *B4GALT1*, and *FTH*, is needed to confirm their reliability and biological relevance. Second, no individual-level phenotype association analysis was performed. Third, the non-cashmere group included both meat and dairy goats, which differ in genetic background, production traits, and breeding history. This grouping may increase within-group variance and reduce the sensitivity of V_ST_ for detecting CNVRs specific to either meat or dairy goats. To reduce this potential bias, pairwise comparisons among cashmere, meat, and dairy goats were also performed as complementary evidence. Future studies with larger and more balanced populations, detailed phenotypic records, and functional experiments are required to further clarify the biological effects of these CNVRs.

## 5. Conclusions

In this study, based on the whole-genome resequencing data of 151 individuals from 17 Chinese goat breeds by the team in the previous period, we conducted a comprehensive study of CNVs in each breed. A total of 5636 CNVRs were identified, covering 2.38% of the whole genome of goats, and 912 protein-coding genes related to CNVR were annotated, and the CNVR map of Chinese goats was constructed. V_ST_ analysis identified several CNVR-associated candidate genes, including *ABCC4*, *APOL3*, *EXOC3L4*, *ERG*, *B4GALT1*, and *FTH*, which may be relevant to production or adaptive trait differentiation in goats. Although these candidate genes should be further validated through functional experiments, our findings provide useful genomic information for exploring molecular markers potentially related to important phenotypic variation in goats.

## Figures and Tables

**Table 1:** CNVR-associated genes showing high divergence between cashmere goats and non-cashmere goats.

Chr	Start	End	Type	V_ST_	Gene Symbol	Description
Chr12	17,816,601	17,818,800	DUP	0.1519	*ABCC4*	Multidrug resistance-associated protein 4
Chr5	73,745,201	73,842,000	DUP	0.2741	*APOL3*	Apolipoprotein L3
Chr21	2,579,001	2,580,200	DEL	0.1949	*EXOC3L4*	Exocyst complex component 3-like protein 4
Chr1	156,173,201	156,178,000	DEL	0.1488	*ERG*	Transcriptional regulator ERG
Chr8	38,003,801	38,005,200	DEL	0.2205	*B4GALT1*	Beta-1,4-galactosyltransferase 1
ChrX	37,652,601	37,660,000	DEL	0.2037	*FTH*	Ferritin heavy chain

## Data Availability

Upon reasonable request, the datasets of this study can be made available by the corresponding author. The raw sequencing data were deposited in the NCBI SRA under accession numbers SRR33322370–SRR33322470 and SRR33515344–SRR33515395.

## Supplementary Materials

The following supporting information can be downloaded at: https://www.mdpi.com/article/10.3390/genes17060627/s1, Figure S1: Histogram of the number distribution of CNVRs on chromosomes; Figure S2: PCA constructed based on total CNVRs; Figure S3: PCA constructed based on DEL CNVRs; Figure S4: PCA constructed based on DUP CNVRs; Figure S5: Manhattan plot of V_ST_ values between cashmere goats and meat goats. The red horizontal line indicates the empirical top 2% V_ST_ threshold; Figure S6: Manhattan plot of V_ST_ values between cashmere goats and dairy goats. The red horizontal line indicates the empirical top 2% V_ST_ threshold; Figure S7: Manhattan plot of V_ST_ values between meat goats and dairy goats. The red horizontal line indicates the empirical top 2% V_ST_ threshold; Table S1: Overview of whole genome resequencing data of 151 goats; Table S2: CNVR data statistics of 151 goats; Table S3: Annotation of CNVRs; Table S4: The overview of the differentiated CNVR between cashmere goats compared to non-cashmere goats; Table S5: List of genes annotated by top2% differential CNVRs of cashmere goats compared to non-cashmere goats; Table S6: KEGG of cashmere goats compared to non-cashmere goats; Table S7: The overview of the differentiated CNVR between cashmere goats compared to meat goats; Table S8: List of genes annotated by top2% differential CNVRs of cashmere goats compared to meat goats; Table S9: The overview of the differentiated CNVR between cashmere goats compared to dairy goats; Table S10: List of genes annotated by top2% differential CNVRs of cashmere goats compared to dairy goats; Table S11: The overview of the differentiated CNVR between meat goats compared to dairy goats. Table S12: List of genes annotated by top2% differential CNVRs of meat goats compared to dairy goats.

## Abbreviations

The following abbreviations are used in this manuscript:

CNV	Copy number variation
CNVR	Copy number variation region
PCA	Principal component analysis
GO	Gene Ontology
KEGG	Kyoto Encyclopedia of Genes and Genomes
