# Supplementary material for: Identification of the Genetic Characteristics of Copy Number Variation Regions in Diverse Goat Populations
Source: Genes (Basel). 2026 May 30;17(6):627. doi: 10.3390/genes17060627 (PMC13298479; doi:10.3390/genes17060627)
Supplement: Supplementary file 1 [file genes-17-00627-s001.zip › Supplementary Figures-revised.pdf]

Supplementary data

File 1: Supplementary Figures

Figure S1. Histogram of the number distribution of CNVRs on chromosomes.

Figure S2. PCA constructed based on total CNVRs.

Figure S3. PCA constructed based on DEL CNVRs.

Figure S4. PCA constructed based on DUP CNVRs.

Figure S5. Manhattan plot of V_{ST} values between cashmere goats and mat goats. The red horizontal line indicates the empirical top 2% V_{ST} threshold.

Figure S6. Manhattan plot of V_{ST} values between cashmere goats and dairy goats. The red horizontal line indicates the empirical top 2% V_{ST} threshold.

Figure S7. Manhattan plot of V_{ST} values between meat goats and dairy goats. The red horizontal line indicates the empirical top 2% V_{ST} threshold.

File 2: Supplementary Tables

Table S1. Overview of whole genome resequencing data of 151 goats.

Table S2. CNVR data statistics of 151 goats.

Table S3. Annotation of CNVRs.

Table S4. The overview of the differentiated CNVR between cashmere goats compared to non-cashmere goats.

Table S5. List of genes annotated by top2% differential CNVRs of cashmere goats compared to non-cashmere goats.

Table S6. KEGG of cashmere goats compared to non-cashmere goats.

Table S7. The overview of the differentiated CNVR between cashmere goats compared to meat goats.

Table S8. List of genes annotated by top2% differential CNVRs of cashmere goats compared to meat goats.

Table S9. The overview of the differentiated CNVR between cashmere goats compared to dairy goats.

Table S10. List of genes annotated by top2% differential CNVRs of cashmere goats compared

to dairy goats.

Table S11. The overview of the differentiated CNVR between meat goats compared to dairy goats.

Table S12. List of genes annotated by top2% differential CNVRs of meat goats compared to dairy goats.

Supplementary Figures:

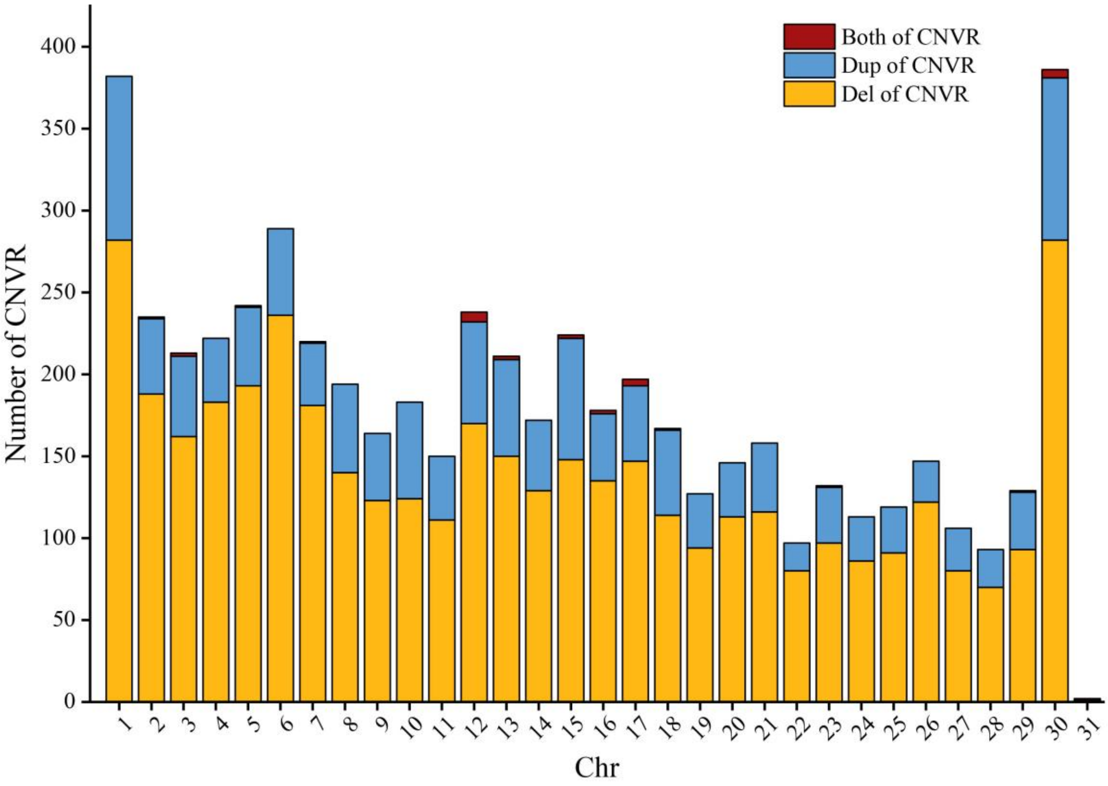

Figure S1. Histogram of the number distribution of CNVRs on chromosomes.

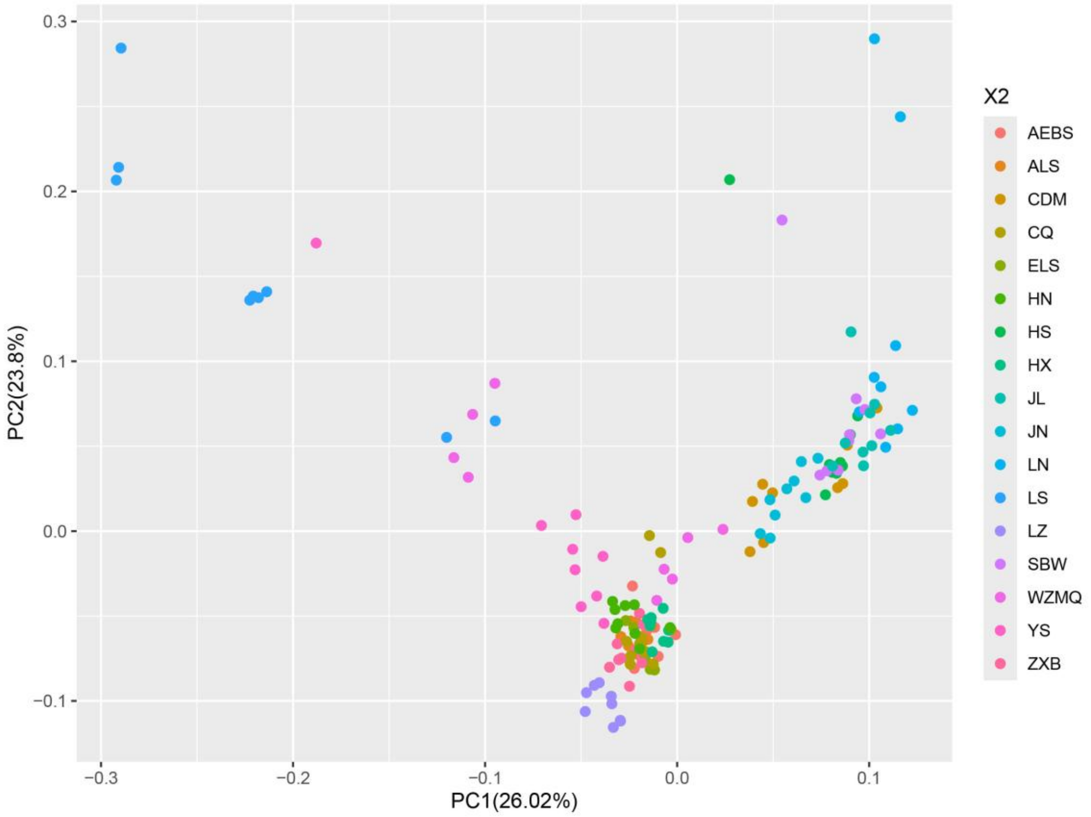

Figure S2. PCA constructed based on total CNVRs.

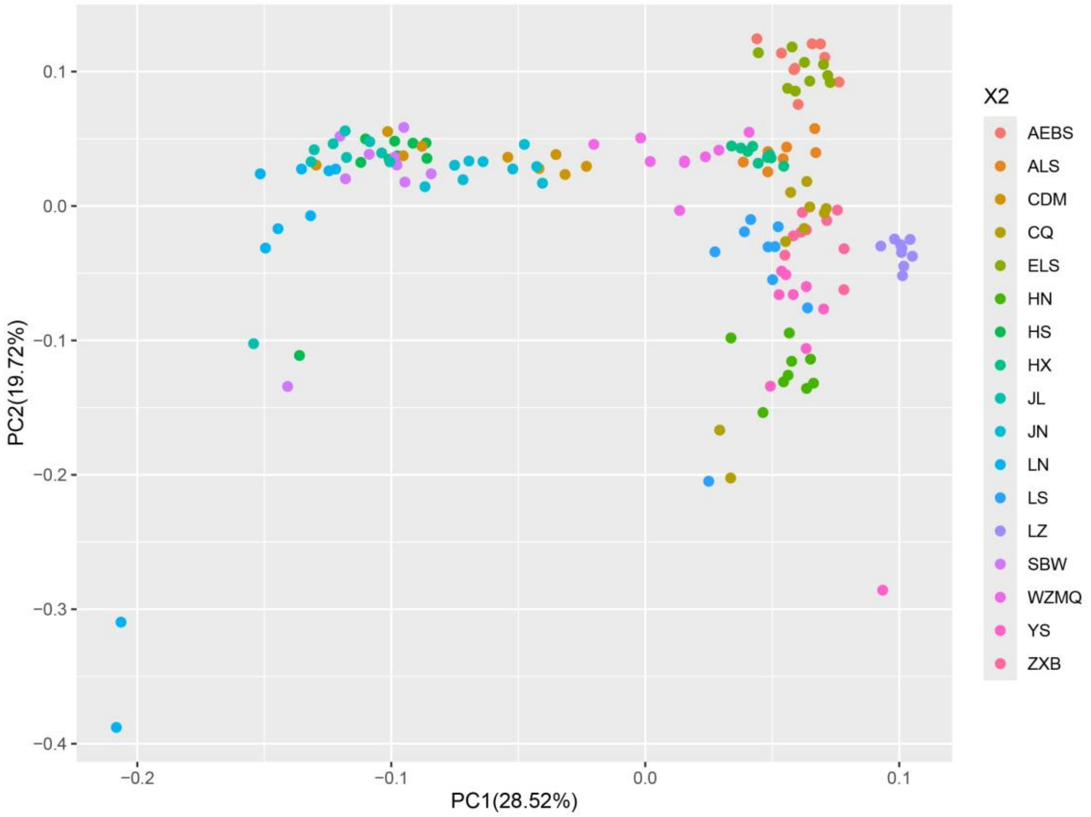

Figure S3. PCA constructed based on DEL CNVRs.

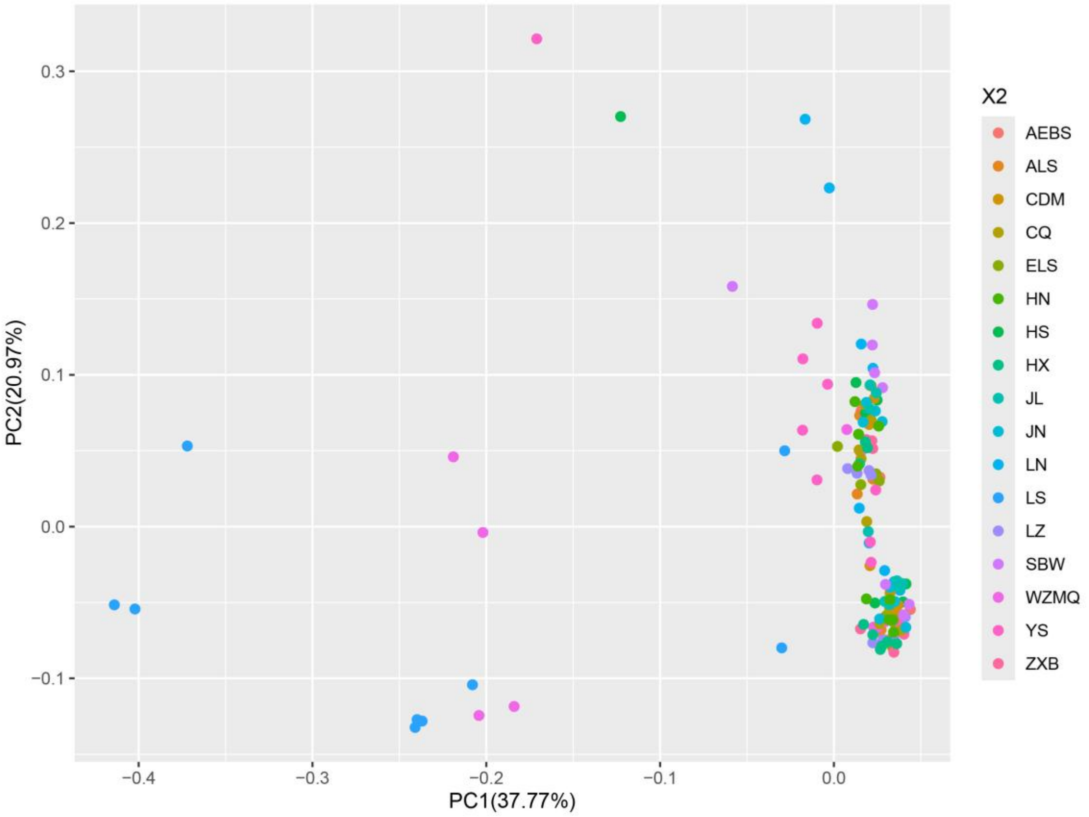

Figure S4. PCA constructed based on DUP CNVRs.

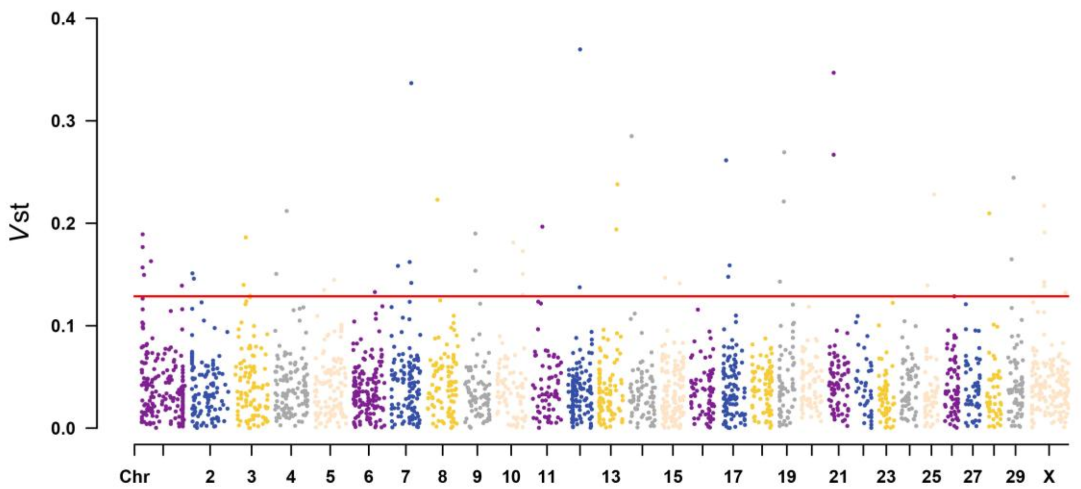

Figure S5. Manhattan plot of V_{ST} values between cashmere goats and mat goats. The red horizontal line indicates the empirical top 2% V_{ST} threshold.

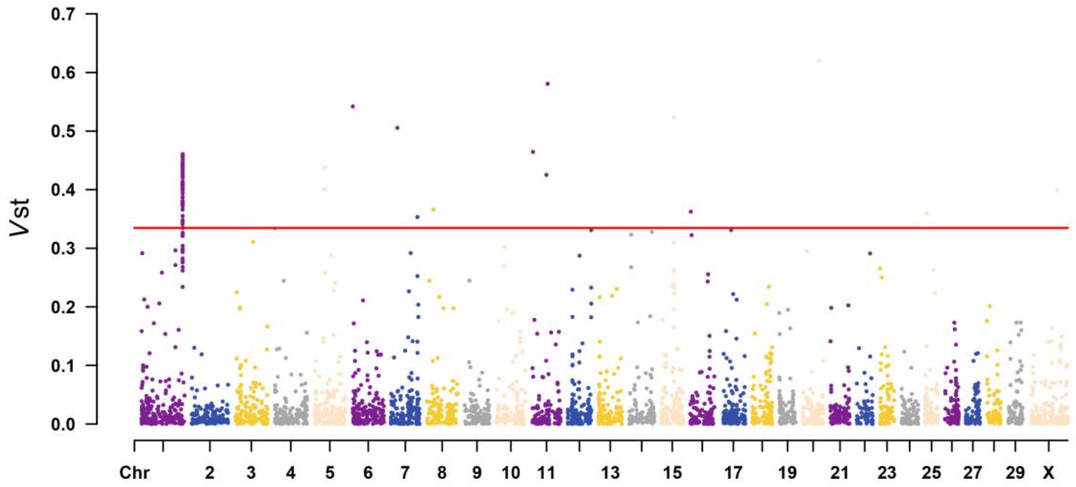

Figure S6. Manhattan plot of V_{ST} values between cashmere goats and dairy goats. The red horizontal line indicates the empirical top 2% V_{ST} threshold.

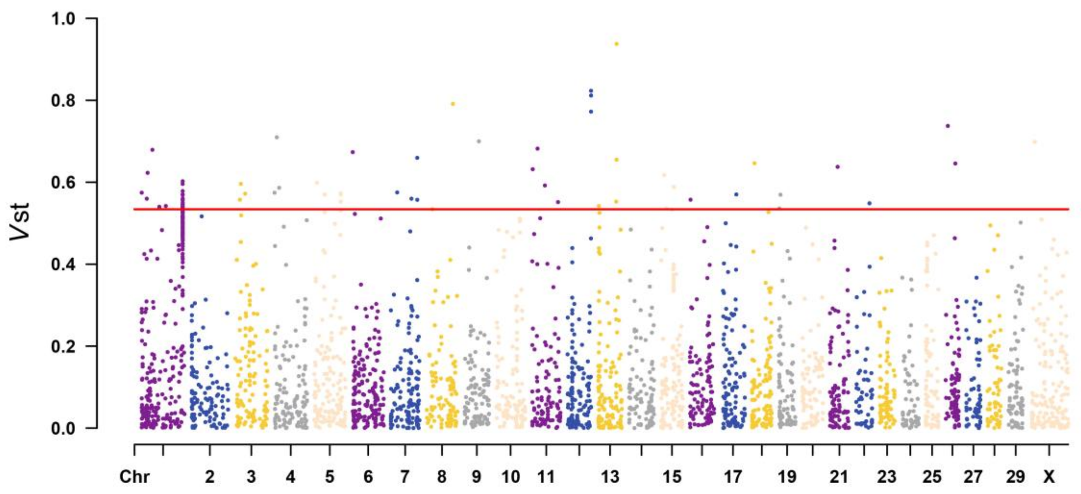

Figure S7. Manhattan plot of V_{ST} values between meat goats and dairy goats. The red horizontal line indicates the empirical top 2% V_{ST} threshold.
